# Socioeconomic Inequalities in Mortality and Repeated Measurement of Explanatory Risk Factors in a 25 Years Follow-Up

**DOI:** 10.1371/journal.pone.0124690

**Published:** 2015-04-08

**Authors:** Věra Skalická, Kristen Ringdal, Margot I. Witvliet

**Affiliations:** Department of Sociology and Political Science, Norwegian University of Science and Technology, Trondheim, Norway; University of Cambridge, UNITED KINGDOM

## Abstract

**Background:**

Socioeconomic inequalities in mortality can be explained by different groups of risk factors. However, little is known whether repeated measurement of risk factors can provide better explanation of socioeconomic inequalities in health. Our study examines the extent to which relative educational and income inequalities in mortality might be explained by explanatory risk factors (behavioral, psychosocial, biomedical risk factors and employment) measured at two points in time, as compared to one measurement at baseline.

**Methods and Findings:**

From the Norwegian total county population-based HUNT Study (years 1984–86 and 1995–1997, respectively) 61 513 men and women aged 25–80 (82.5% of all enrolled) were followed-up for mortality in 25 years until 2009, employing a discrete time survival analysis. Socioeconomic inequalities in mortality were observed. As compared to their highest socioeconomic counterparts, the lowest educated men had an OR (odds ratio) of 1.41 (95% CI 1.29–1.55) and for the lowest income quartile OR = 1.59 (1.48–1.571), for women OR = 1.35 (1.17–1.55), and OR = 1.40 (1.28–1.52), respectively. Baseline explanatory variables attenuated the association between education and income with mortality by 54% and 54% in men, respectively, and by 69% and 18% in women. After entering time-varying variables, this attainment increased to 63% and 59% in men, respectively, and to 25% (income) in women, with no improvement in regard to education in women. Change in biomedical factors and employment did not amend the explanation.

**Conclusions:**

Addition of a second measurement for risk factors provided only a modest improvement in explaining educational and income inequalities in mortality in Norwegian men and women. Accounting for change in behavior provided the largest improvement in explained inequalities in mortality for both men and women, as compared to measurement at baseline. Psychosocial factors explained the largest share of income inequalities in mortality for men, but repeated measurement of these factors contributed only to modest improvement in explanation. Further comparative research on the relative importance of explanatory pathways assessed over time is needed.

## Introduction

Socioeconomic inequalities in mortality can to a certain extent be explained by variation in health related risk factors. According to current theoretical explanations, these risk factors are related to material, psychosocial, behavioral and known biomedical/biological groups of factors [1–4]. In previous research, several studies quantified the contribution of the respective risk factors to help explain social inequalities in health. Findings ranged from 12% to 54% for behavioral factors [3, 5, 6] and 73% to 83% for a combination of all above mentioned factors [4]. In most studies, however, the risk factors have only been assessed at one time point.

However, according to previous research, health behavior and other risk factors can change over time and this change might be socially patterned [7–11]. For example, socioeconomically disadvantaged groups might have limited resources to adopt healthy lifestyle, their mental health may deteriorate over time due to experiencing more adverse life circumstances, their social network might be less supportive and their physical health might be more influenced by lasting unhealthy behavior as compared to socioeconomically affluent groups [7–11].

In addition, mortality, health behavior and risk factor differences between men and women may also be apparent [11–13]. Men are more likely than women to have unfavorable levels of risk behaviors, e.g. heavy drinking [14, 15]). On the contrary, women show higher levels of obesity [16]. Furthermore, change in behavior may also be gender specific: in 1980`s, Norwegian men had higher prevalence in smoking than women; however, over time, men’s prevalence of smoking declined, while women experienced an increase in smoking in all socioeconomic groups [17].

Given changes in health behavior over the life course, it is plausible that assessment of change in risk factors over time might provide more accurate information about the varying impact of explanatory mechanisms [18]. For example, assessment of time-varying behavior risk factors might result in a larger part of social inequalities in mortality being explained, as compared to measurement at baseline only [19], but the magnitude of the explained share might vary according to cultural context and social patterning of risk factors [20]. Previous studies have assessed how change in behavioral [19], psychosocial [21, 22], and biomedical factors [8, 23] and employment [24] affect explanation of socioeconomic inequalities in health. However, to the best of our knowledge, no study has compared how accounting for change over time of different groups of explanatory factors together can contribute to explanation of social inequalities in mortality.

The aim of this study is thus to compare to what extent can education and income inequalities in mortality be explained by four groups of explanatory factors, (e.g. behavioral, psychosocial, biomedical factors and employment status) in men and women separately, assessed at two time points as compared to baseline.

## Methods

We combined data from two waves of the Norwegian total county population HUNT 1 (1984–86) and HUNT 2 Study (1995–97) [25]. The HUNT study has been considered to be generally representative for the Norway as a whole [26]. In HUNT 1, all residents of the Nord-Trøndelag county aged 20 and over were approached. Number of respondents was 74 599 (88.1% of the adult population). We included persons aged 25 to 80 years in the current analyses. The lower age limit was set in order to have respondents’ completed education recorded. People with missing information on education were excluded (n = 398), and those who reported history of health problems (i.e. diabetes, angina pectoris, stroke, cerebral hemorrhage and heart attack, n = 5 743). These diseases were assessed as a simple question in the health survey. By selecting relatively healthy people for the analysis, we aim to reduce the possibility of reverse causality, given that sick people might change their behaviour. This left a sample of 29 776 men and 31 747 women, which provided information for the baseline risk factors measured in 1984–86. From this sample, 22 781 men and 25 536 women participated also in HUNT 2 and provided information on explanatory risk variables at the second time point (1995–97). During the mortality follow-up period (1984 to December 31^st^, 2009) 10 267 men and 9 404 women died. In the period 1984–1995, 3 748 men and 2 687 women died and in the period 1996–2009, 6 519 men and 6 717 women died. The linkage of the sample to the national death registry [27], together with data on education (collected in 1970–1985) and income (collected in 1980–1985)—which were measured only at baseline—was provided and administrated by Statistics Norway. The linkage between the data sources was made possible by a unique personal identity number, which is given to every citizen in Norway. These linkages have been approved by the Norwegian Data Inspectorate and the current study was approved by the Regional Committee for Medical Research Ethics in Middle Norway. All participants signed a written consent to take part in the study, and have a standing opportunity to withdraw their consent at any time. The data can be applied for at the NTNU HUNT research center [25].

### Socioeconomic variables

The most often used measures of socioeconomic position include education, occupation and income [9]. Since 34% of women could not be classified according to the EGP occupational classification, we focused only on education and income. These measures are related, but refer to distinct aspects of socioeconomic position and their relation to mortality might tap in different causal mechanisms [28]. By employing both measures in the analysis, one might be able to compare how different aspects of socioeconomic position affect mortality through presumably different pathways.

We used the Norwegian national education data base (NUDB) as the primary source for the highest education attained rather than the HUNT data. The reason for this was the high number of cases of missing data in HUNT 1, attributable to a high rate of non-response to part of the questionnaire including the question on education, which was to be returned by postal mail. The indicator was based on the national census in 1970 and subsequent information from schools updated until 1985. We reclassified the education data into three levels—primary (< = 9 years) and lower secondary (10–11 years), upper secondary (12 years), and tertiary (13 +; reference category). Wherever possible, missing NUDB data on education were replaced by information on education from the HUNT questionnaire (n = 913). However, we had no information on education from either NUDB or HUNT (n = 398), and these persons were excluded from the analysis.

The income variable stems from Norwegian tax authorities`data on individual pensionable income and was calculated as the average of three subsequent data on income (years 1980, 1984 and 1985). Income quartiles were created separately for men and women, based on income distribution in the whole HUNT survey, as the income distribution is dissimilar between sexes. The lowest quartile category comprises also people with no income and retired pensioners without additional income. The quartile with the highest income was the reference category (quartile IV).

### Risk factors

Behavioural factors were measured by information about smoking (more or less than 20 cigarettes per day, being a former smoker, never smoked), alcohol consumption (moderate: 0–4 times in 2 weeks, extensive: more than 4 times in 2 weeks, abstinent) and physical activity.

Physical activity measured in two questions was scored with number of hours per week spent on activity type: hard physical activity was given twice as much weight as slight physical activity (very active: 6–9 hours/week, inactive: 0–1 hour/week, moderate: 2–5 hours/week). Persons missing information on either hard or slight physical activity were assigned the modus value of activity typical for persons who performed the same amount of the non-missing activity.

Based on psychological models, which integrate stress and resources into explanatory pathways of the SES—health relation (9), following psychosocial measures of social support and negative emotions/well-being were included: civil status (married, single, divorced/separated, widowed), feeling lonely (1 = very often, often, 0 = sometimes, seldom, never at T1 and 1 = not having enough good friends, 0 = having enough good friends at T2), and three measures on a 7 item Likert scale: feeling tired (0 = 1 through 3, 1 = 4 through 7; 7 = very tired), feeling unsatisfied (0 = 1 through 4, 1 = 5 through 7; 7 = very unsatisfied) and feeling unhappy (0 = 1 through 3, 1 = 4 through 7; 7 = very unhappy). Example of a question (for tiredness) is: “On the whole, do you feel strong and in a good mood, or tired and exhausted?”

Employment status was coded in four categories (1. labour force, 2. homemaker, 3. retired, 4. unemployed). The last category (4.) also included low prevalent categories of military service/education.

Biomedical factors included BMI measured in three categories (<20, 20–30, > = 30 kg/m²), and hypertension, defined as having systolic blood pressure >140 mmHg and/or diastolic blood pressure >90 mmHg.

### Statistical analyses

Discrete time survival analysis [29] was applied to analyse mortality data, where survival times were grouped into discrete intervals of time (years). This data set up makes it possible to include change in time-varying variables. Age adjusted odds ratios (OR) for levels of education and income, respectively, were calculated by means of discrete time logistic model separately for men and women, since there was a significant interaction between income and gender. Although there was no significant interaction between gender and education, we also conducted separate analysis of education by gender for reasons of consistency. All analyses were thus conducted for men and women separately. ORs were calculated for men and women aged 25–80 years and also for two age subgroups (25–59 and 60–80 years). Age adjusted mortality rates per 100 000 person-years employing direct standardization in 5 years age intervals for each level of socioeconomic position were calculated separately for two age groups (25–59 years and 60–80 years). Age adjusted ORs were calculated for all baseline risk factors separately, adjusted first for education and second for income, and age standardized differences in risk factor prevalence between primary and tertiary educated and between lowest and highest income quartile were estimated, employing 10 years age intervals.

In the main analysis, we used the above mentioned associations between education and income and mortality (ORs and their 95% confidence intervals), adjusted for age, as a reference model. In all regression analyses, age was accounted for as a continuous measure and as age squared. Next, we adjusted for behavioural, psychosocial, and biomedical factors (and employment for education) separately, followed by adjustment for all explanatory factors simultaneously—first for baseline explanatory factors, and second including repeated measurement. For each model, the percentage change in ORs of each level of education and income, respectively, was calculated [4]. The fit of the models were assessed using -2LogLikelihood test and the AIC and BIC criteria. The analyses were done for men and women aged 25–80 years. The analyses were run using STATA MP 11.2.

We conducted also several sets of sensitivity analyses. First, we repeated the main analysis for men and women aged 25–59 and 60–80 years, respectively. Second, we extended the main analysis to respondents, who were originally excluded due to history of disease, while controlling for morbidity. Third, we excluded persons who died before the second survey. Finally, we also conducted another analysis restricted only to respondents with complete data.

## Results

Education and income were associated with mortality and followed a stepwise gradient. In men, OR for mortality was 1.41 (1.29–1.55) for primary education and 1.29 (1.41–1.45) for secondary education and for lowest through second highest income quartile ORs were 1.59 (1.48–1.71), 1.31 (1.22–1.42) and 1.17 (1.08–1.26) respectively (Table 1). In women, only lowest education OR 1.35 (1.17–1.55) and lowest and next lowest income quartile with OR 1.40 (1.28–1.52) and 1.15 (1.04–1.27), respectively, showed significantly greater OR as compared to their reference categories (Table 2). Education and income inequalities in mortality were generally smaller in older people (60–80 years) than in younger ages (25–59 years) (Table 1 and 2).

**Table 1:** Association between education and mortality and income and mortality, 29,766 men 25–80 years, 1984/86–2009.

	Men 25–80 years					Men 25–59 years			Men 60–80 years		
	No. men	No. deaths	Mean age	OR	CI 95%	Mortality rate	OR	CI 95%	Mortality rate	OR	CI 95%
**Education**											
Primary	21689	9031	51	**1.41**	(1.29–1.55)	557	**1.56**	(1.38–1.78)	831	**1.23**	(1.08–1.41)
Secondary	4464	651	37	**1.29**	(1.14–1.45)	485	**1.34**	(1.14–1.58)	834	**1.21**	(1.01–1.45)
Tertiary	3613	585	42	1.00		399	1.00		771	1.00	
**Income**											
I. inc.quartile	7737	5526	61	**1.59**	(1.48–1.71)	737	**2.02**	(1.82–2.24)	868	**1.42**	(1.27–1.59)
II. inc. quartile	6539	1693	43	**1.31**	(1.22–1.42)	555	**1.38**	(1.25–1.52)	794	**1.14**	(1.01–1.28)
III. inc. quartile	7667	1601	43	**1.17**	(1.08–1.26)	510	**1.20**	(1.09–1.32)	736	1.05	(0.92–1.20)
IV. inc. quartile (highest)	7823	1447	44	1.00		439	1.00		740	1.00	

Note: OR = odds ratio CI = confidence interval

OR was adjusted for age and age squared.

Age adjusted mortality rate / 100 000 person years was standardized by means of direct standardization (World standard population).

Values in bold do not include the OR 1.00.

**Table 2:** Association between education and mortality and income and mortality, 31,747 women 25–80 years, 1984/86–2009.

	Women 25–80 years					Women 25–59 years			Women 60–80 years		
	No. women	No. deaths	Mean age	OR	CI 95%	Mortality rate	OR	CI 95%	Mortality rate	OR	CI 95%
**Education**											
Primary	26416	8837	51	**1.35**	(1.17–1.55)	354	**1.42**	(1.17–1.73)	638	**1.24**	(1.17–1.55)
Secondary	2624	317	39	1.09	(0.91–1.30)	292	1.09	(0.83–1.41)	599	1.06	(0.83–1.35)
Tertiary	2707	250	39	1.00		241	250	39	596		
**Income**											
I. inc.quartile	12693	6666	57	**1.40**	(1.28–1.52)	446	**1.66**	(1.48–1.87)	649	**1.17**	(1.03–1.34)
II.inc. quartile	6026	1152	42	**1.15**	(1.04–1.27)	339	**1.18**	(1.03–1.35)	621	1.01	(0.87–1.18)
III.inc.quartile	6340	795	43	1.01	(0.91–1.12)	296	1.03	(0.91–1. 18)	612	0.93	(0.79–1.11)
IV.inc.quartile (highest)	6688	791	43	1.00		283	1.00		512	1.00	

Note: OR = odds ratio CI = confidence interval

OR was adjusted for age and age squared.

Age adjusted mortality rate / 100 000 person years was standardized by means of direct standardization (World standard population).

Values in bold do not include the OR 1.00.

All risk factors increased risk of mortality for men and women, after adjustment for either education or income—with a few exceptions. In men, categories of homemaking and abstainers were not significantly different from their respective reference categories. Moderate exercise in men with OR 0.93 (0.86–0.99) showed to be protective, compared to very active when controlled for education, while there was no significant difference between those two categories when adjusted for income (Table 3). In women, moderate exercise did not increase the risk of mortality, compared to very active (both by education and income). When adjusted for income, neither abstinence, widowhood, unemployment nor homemaking in women was significantly different from their respective reference categories in respect to mortality (Table 4).

**Table 3:** Age-adjusted bivariarite impacts (odds ratios with 95% confidence intervals) on mortality of employment, three behavioural, five psychosocial and two biomedical factors at baseline, controlling first for education (Column 1) and second for income (Column 2).

**Employment status**	Education		Income	
**Employment**				
Unemployed/military/education	**1.58**	(1.40–1.78)		
Retired/social benefits	**1.38**	(1.30–1.46)		
Homemaker	1.15	(0.89–1.47)		
Missing	1.30	(0.85–1.97)		
In labour force	1.00			
**Behavioural factors**	Education		Income	
**Smoking**				
Smoker > = 20 cig.	**3.38**	(3.08–3.72)	**3.36**	(3.06–3.69)
Smoker < 20 cig.	**1.78**	(1.67–1.90)	**1.79**	(1.68–1.91)
Former smoker	**1.13**	(1.06–1.21)	**1.15**	(1.07–1.23)
Missing	**1.49**	(1.40–1.60)	**1.50**	(1.40–1.60)
Never smoker	1.00		1.00	
**Physical activity**				
Inactive (0–1 h)	**1.13**	(1.06–1.20)	**1.16**	(1.09–1.24)
Moderately active (2–5 h)	**0.93**	(0.86–0.99)	0.95	(0.88–1.02)
Missing	**1.26**	(1.17–1.36)	**1.29**	(1.19–1.39)
Active (6–9 h)	1.00		1.00	
**Alcohol**				
Extensive (5+ times in 2 weeks)	**1.16**	(1.07–1.25)	**1.15**	(1.07–1.24)
Abstinent	1.03	(0.95–1.11)	1.01	(0.93–1.09)
Missing	**1.28**	(1.21–1.36)	**1.27**	(1.20–1.35)
Moderate (0–4 times in 2 weeks)	1.00		1.00	
Table 2A cont.				
**Psychosocial factors**	Education		Income	
**Feeling lonely**				
Feeling lonely	**1.42**	(1.29–1.58)	**1.38**	(1.24–1.52)
Missing	**1.26**	(1.19–1.33)	**1.25**	(1.18–1.32)
Not feeling lonely	1.00		1.00	
Feeling tired				
**Feeling tired**	**1.35**	(1.29–1.41)	**1.32**	(1.26–1.38)
Missing	**1.42**	(1.34–1.51)	**1.40**	(1.32–1.49)
Feeling strong	1.00		1.00	
**Feeling unsatisfied**				
Feeling unsatisfied	**1.13**	(1.07–1.19)	**1.12**	(1.06–1.17)
Missing	**1.29**	(1.22–1.37)	**1.28**	(1.21–1.36)
Feeling satisfied	1.00		1.00	
**Feeling unhappy**				
Unhappy	**1.35**	(1.28–1.42)	**1.30**	(1.23–1.38)
Missing	**1.22**	(1.03–1.45)	**1.20**	(1.01–1.43)
Happy	1.00		1.00	
**Civil status**				
Unmarried	**1.20**	(1.11–1.31)	**1.39**	(1.31–1.48)
Separated	**1.40**	(1.23–1.60)	**1.56**	(1.39–1.75)
Widowed	1.02	(0.97–1.08)	**1.21**	(1.11–1.33)
Missing	1.17	(0.76–1.80)	0.92	(0.57–1.51)
Married	1.0			
**Biomedical factors**	Education		Income	
**BMI**				
BMI < 20	**1.53**	(1.35–1.73)	**1.48**	(1.30–1.67)
BMI > = 30	**1.12**	(1.07–1.17)	**1.12**	(1.07–1.17)
Missing	1.18	(1.00–1.39)	1.17	(1.00–1.38)
BMI 20–29.9	1.00		1.00	
**Hypertension**				
Sys >140 or dias > 90	**1.23**	(1.18–1.29)	**1.23**	(1.18–1.28)
Sys < = 140 or dias < = 90	1.00		1.00	

29,766 men aged 25–80 years.

Note: Adjusted for age and age squared.

Values in bold do not include the OR 1.00.

**Table 4:** Age-adjusted bivariarite impacts (odds ratios with 95% confidence intervals) on mortality of employment, three behavioural, five psychosocial and two biomedical factors at baseline, controlling first for education (Column 1) and second for income (Column 2).

**Employment status**	Education		Income	
**Employment**				
Unemployed/military/education	**1.17**	(1.02–1.33)		
Retired/social benefits	**1.50**	(1.39–1.60)		
Homemaker	**1.21**	(1.13–1.30)		
Missing	**1.44**	(1.03–2.02)		
In labour force	1.00			
**Behavioural factors**	Education		Income	
**Smoking**				
Smoker > = 20 cig.	**4.43**	(3.86–5.08)	**4.46**	(3.89–5.12)
Smoker < 20 cig.	**1.82**	(1.70–1.94)	**1.84**	(1.72–1.96)
Former smoker	**1.10**	(1.01–1.21)	**1.11**	(1.01–1.21)
Missing	**1.24**	(1.18–1.31)	**1.25**	(1.18–1.31)
Never smoker	1.00		1.00	
**Physical activity**				
Inactive (0–1 h)	**1.22**	(1.12–1.32)	**1.22**	(1.13–1.33)
Moderately active (2–5 h)	0.97	(0.89–1.06)	0.97	(0.89–1.06)
Missing	**1.39**	(1.27–1.52)	**1.41**	(1.28–1.54)
Active (6–9 h)	1.00		1.00	
**Alcohol**				
Extensive (5+ times in 2 weeks)	**1.15**	(1.00–1.33)	**1.19**	(1.13–1.27)
Abstinent	**1.07**	(1.01–1.14)	1.06	(1.00–1.12)
Missing	**1.19**	(1.13–1.27)	1.13	(0.98–1.31)
Moderate (0–4 times in 2 weeks)	1.00		1.00	
**Psychosocial factors**	Education		Income	
**Feeling lonely**				
Feeling lonely	**1.21**	(1.12–1.31)	**1.19**	(1.10–1.29)
Missing	**1.26**	(1.18–1.34)	**1.27**	(1.19–1.35)
Not feeling lonely	1.00		1.00	
**Feeling tired**				
Feeling tired	**1.29**	(1.23–1.35)	**1.27**	(1.21–1.33)
Missing	**1.41**	(1.32–1.51)	**1.41**	(1.32–1.51)
Feeling strong	1.00		1.00	
**Feeling unsatisfied**				
Feeling unsatisfied	**1.17**	(1.11–1.23)	**1.15**	(1.10–1.21)
Missing	**1.29**	(1.21–1.37)	**1.29**	(1.22–1.38)
Feeling satisfied	1.00		1.00	
**Feeling unhappy**				
Unhappy	**1.32**	(1.25–1.40)	**1.30**	(1.22–1.37)
Missing	**1.45**	(1.23–1.70)	**1.42**	(1.21–1.67)
Happy	1.00		1.00	
**Civil status**				
Unmarried	**1.47**	(1.39–1.56)	**1.22**	(1.13–1.33)
Separated	**1.62**	(1.44–1.82)	**1.42**	(1.25–1.62)
Widowed	**1.24**	(1.13–1.35)	1.03	(0.97–1.08)
Missing	1.02	(0.63–1.66)	1.14	(0.74–1.76)
Married	1.0			
**Biomedical factors**	Education		Income	
**BMI**				
BMI < 20	**1.56**	(1.40–1.75)	**1.53**	(1.38–1.72)
BMI > = 30	**1.11**	(1.05–1.16)	**1.09**	(1.04–1.14)
Missing	**1.71**	(1.46–2.01)	**1.71**	(1.46–2.00)
BMI 20–29.9	1.00		1.00	
**Hypertension**				
Sys >140 or dias > 90	**1.25**	(1.19–1.31)	**1.24**	(1.18–1.29)
Sys < = 140 or dias < = 90	1.00		1.00	

31,747 women aged 25–80 years.

Note: Adjusted for age and age squared.

Values in bold do not include the OR 1.00.

In men, largest differences in prevalence of risk factors between lowest and highest education were in physical activity and smoking, while with regard to prevalence differences between lowest and highest income, differences in psychosocial factors (unmarried, unhappiness and tiredness) were most pronounced. Over time, differences in prevalence according to education increased or remained the same for all factors, except for physical activity, hypertension, loneliness, unhappiness and tiredness, which decreased. Differences by income decreased or remained on the same level, with an increase in differences by former smoking, inactivity and abstinence (Table 5). In women, largest prevalence differences between lowest and highest education were in homemakers, moderate smoking, physical activity and obesity. The largest prevalence differences between women with lowest and highest income were in tiredness and obesity. Over time, differences in prevalence according to education decreased or remained the same, with an increase in moderate smoking, extensive alcohol (reverted gradient), unhappiness, widowhood, obesity and hypertension. Prevalence differences in women by income also decreased over time, with a few exceptions in increase in heavy and former smoking, physical activity and widowhood (Table 6).

**Table 5:** Differences in age-standardized prevalence (%) of high-risk categories between high and low education and between highest and lowest income quartile, first at baseline and second at follow up, men.

	Education		Income	
	Baseline	Follow up	Baseline	Follow up
**Employment status**				
Homemaker	0	0		
Unemployed	2	2		
Retired	5	6		
**Behavioural factors**				
**Smoking**				
Smoker > = 20 cig.	2	3	3	1
Smoker < 20 cig.	11	12	6	2
Former smoker	-2	2	-7	6
**Physical activity**				
Inactive (0–1 h)	14	8	5	8
Moderat. active (2–5 h)	-17	-7	-10	-6
**Alcohol**				
Extensive (>14 drinks)	-3	-7	5	-3
Abstinent	-3	0	3	6
**Psychosocial factors**				
**Feeling lonely**				
Lonely	1	-5	4	-1
**Feeling tired**				
Tired	8	6	14	9
**Feeling unsatisfied**				
Unsatisfied	6	2	9	8
**Feeling unhappy**				
Unhappy	6	5	16	9
**Civil status**				
Unmarried	5	6	27	26
Separated	0	1	2	1
Widowed	1	1	1	1
**Biomedical factors**				
**BMI**				
BMI < 20	0	0	3	2
BMI> = 30	8	8	1	-2
**Hypertension**				
Sys >140 or dias > 90	6	7	5	-1

Note: Standardized by the direct method.

All changes in prevalence differences were statistically significant (t-test).

**Table 6:** Differences in age-standardized prevalence (%) of high-risk categories between high and low education and between highest and lowest income quartile, first at baseline and second at follow up, women.

	Education		Income	
	Baseline	Follow up	Baseline	Follow up
**Employment status**				
Homemaker	26	12		
Unemployed	2	1		
Retired	5	3		
**Behavioural factors**				
**Smoking**				
Smoker > = 20 cig.	1	1	0	30
Smoker < 20 cig.	14	15	3	2
Former smoker	-3	-2	1	2
**Physical activity**				
Inactive (0–1 h)	11	8	6	8
Moderat. active (2–5 h)	-15	-7	-3	-4
**Alcohol**				
Extensive (>14 drinks)	-2	-5	-1	-1
Abstinent	-3	1	-1	7
**Psychosocial factors**				
**Feeling lonely**	2	-4	4	2
Lonely				
**Feeling tired**				
Tired	7	5	13	5
**Feeling unsatisfied**				
Unsatisfied	5	1	9	5
**Feeling unhappy**				
Unhappy	3	5	9	5
**Civil status**				
Unmarried	-5	-3	-8	-7
Separated	1	1	-2	-2
Widowed	4	6	3	4
**Biomedical factors**				
**BMI**				
BMI < 20	-1	-1	1	1
BMI> = 30	10	13	10	6
**Hypertension**				
Sys >140 or dias > 90	9	12	9	6

Note: Standardized by the direct method.

All changes in prevalence differences were statistically significant (t-test).

In men, behavioural and psychosocial factors explained the largest proportion of mortality risk for low educated at the baseline (27% and 24%, respectively). When we accounted for change of these factors over time, explained proportion reached 39% and 29% respectively. All groups of risk factors explained together 54% of the excess mortality risk in low educated, and 63% when accounting for change over time (Table 7). In income inequalities in mortality, psychosocial factors explained a larger share than behaviour (41% versus 19%). However, over time, change in psychosocial factors improved the explanation of income inequalities in health by only 1%, while change in behaviour amended the explanation by 8%. When change in all explanatory factors was accounted for, 59% of excess mortality in low income group was explained, compared to 54% by all explanatory factors at baseline (Table 8). The AIC/BIC statistics suggested that models accounting for change in biomedical factors and employment had worse fit than models employing baseline measurement, whereas the fit of all the other models accounting for change was better compared to baseline models (Table 7 and 8).

**Table 7:** Odds ratios and proportional change for mortality by education in men 25–80 years.

	Education Baseline						Education Follow up						Change difference
Model	OR	95% CI	% change	-2LL	AIC	BIC	OR	95% CI	% change	-2LL	AIC	BIC	
Age adjusted	1.41	(1.29–1.55)		39 073	78 206	78 546							
Employment	1.37	(1.25–1.51)	10	38 996	78 060	78 445	1.38	(1.26–1.51)	7	39 036	78 142	78 538	-3
Behavioural	1.30	(1.18–1.43)	27	38 622	77 327	77 791	1.25	(1.14–1.38)	39	38 505	77 093	77 557	12
Psychosocial	1.31	(1.20–1.44)	24	38 848	77 780	78 225	1.29	(1.18–1.42)	29	38 809	77 703	78 179	5
Biomedical	1.39	(1.27–1.53)	5	39 001	78 071	78 456	1.40	(1.27–1.53)	2	39 032	78 134	78 530	-3
All	1.19	(1.08–1.31)	54	38 360	76 843	77 534	1.15	(1.04–1.26)	63	38 288	76 702	77 416	9

Note: OR for low education compared to high education. Adjusted for age and age squared.

All nested models were significantly improved based on the -2 Log Likelihood (-2LL) test

AIC = Akaike information criterion BIC = Bayesian information criterion

**Table 8:** Odds ratios and proportional change for mortality by income in men 25–80 years.

	Income Baseline						Income Follow up						Change difference
Model	OR	95% CI	% change	-2LL	AIC	BIC	OR	95% CI	% change	-2LL	AIC	BIC	
Age adjusted	1.59	(1.48–1.71)		39 021	78 104	78 455							
Behavioural	1.48	(1.38–1.60)	19	38 577	77 239	77 715	1.43	(1.33–1.54)	27	38 466	77 017	77 493	8
Psychosocial	1.35	(1.25–1.46)	41	38 833	77 753	78 240	1.34	(1.24–1.44)	42	38 795	77 677	78 164	1
Biomedical	1.56	(1.45–1.68)	5	38 953	77 976	78 372	1.57	(1.45–1.69)	3	38 981	78 035	78 443	-2
All	1.27	(1.18–1.38)	54	38 377	76 871	77 528	1.24	(1.15–1.34)	59	38 294	76 707	77 375	5

Note: OR for lowest income quartile compared to highest income quartile. Adjusted for age and age squared.

All nested models were significantly improved based on the -2 Log Likelihood (-2LL) test.

AIC = Akaike information criterion BIC = Bayesian information criterion

In women, behavioural (49%) and employment factors (17%) explained most of the excess mortality risk in low educated, and all risk factors together explained 69%. However, when accounting for change in all the risk factors, explained proportion of excess mortality reached only 57%. (Table 9). Both employment (-8%) and biomedical factors (-17%) contributed to such decrease. This was also confirmed by the AIC/BIC statistics suggesting worse fit of the models accounting for change in employment and biomedical factors, as compared to models with respective baseline measurements (Table 9). However, the fit of the model accounting for change in all risk factors had still better fit than the baseline model (Table 9). Mortality risk of lowest income in women could be best explained by biomedical factors (10%) at baseline, and by behavioural (20%) and psychosocial factors (10%) at follow up. Behaviour provided the largest increase (17%) in explanation at follow-up, compared to baseline. All baseline factors together explained only 18% of income inequalities in mortality, and 25% when accounting for change (Table 10). Models accounting for change had better fit than the baseline models according to AIC/BIC, except for the model employing biomedical factors (Table 10).

**Table 9:** Odds ratios and proportional change for mortality by education in women 25–80 years.

	Education Baseline						Education Follow up						Change difference
Model	OR	95% CI	% change	-2LL	AIC	BIC	OR	95% CI	% change	-2LL	AIC	BIC	
Age adjusted	1.35	(1.17–1.55)		35 870	71 800	72 143							
Employment	1.29	(1.12–1.48)	17	35 805	71 678	72 066	1.32	(1.15–1.52)	9	35 857	71 785	72 185	-8
Behavioural	1.18	(1.02–1.36)	49	35 502	71 086	71 555	1.16	(1.01–1.34)	54	35 433	70 949	71 417	5
Psychosocial	1.32	(1.15–1.51)	9	35 750	71 584	72 064	1.31	(1.14–1.50)	11	35 728	71 540	72 020	2
Biomedical	1.31	(1.14–1.51)	11	35 777	71 622	72 011	1.37	(1.19–1.57)	-6	35 779	71 629	72 029	-17
All	1.11	(0.96–1.28)	69	35 314	70 750	71 447	1.15	(1.00–1.33)	57	35 295	70 717	71 437	-12

Note: OR for low education compared to high education. Adjusted for age and age squared.

All nested models were significantly improved based on the -2 Log Likelihood (-2LL) test

AIC = Akaike information criterion BIC = Bayesian information criterion

**Table 10:** Odds ratios and proportional change for mortality by income in women 25–80 years.

	Income Baseline						Income Follow up						Change difference
Model	OR	95% CI	% change	-2LL	AIC	BIC	OR	95% CI	% change	-2LL	AIC	BIC	
Age adjusted	1.40	(1.28–1.52)		35 836	71 734	72 088							
Behavioural	1.39	(1.27–1.51)	3	35 462	71 009	71 489	1.32	(1.21–1.44)	20	35 403	70 891	71 370	17
Psychosocial	1.38	(1.27–1.51)	5	35 722	71 530	72 021	1.36	(1.25–1.48)	10	35 702	71 490	71 982	5
Biomedical	1.36	(1.24–1.48)	10	35 749	71 568	71 968	1.39	(1.28–1.52)	3	35 749	71 570	71 981	-7
All	1.33	(1.22–1.45)	18	35 321	70 759	71 422	1.30	(1.19–1.42)	25	35 279	70 677	71 351	7

Note: OR for lowest income quartile compared to highest income quartile. Adjusted for age and age squared.

All nested models were significantly improved based on the -2 Log Likelihood (-2LL) test

AIC = Akaike information criterion BIC = Bayesian information criterion

### Additional Analyses

In the first sensitivity analysis, we found that in people under 60 years of age, baseline factors accounted for larger explanation of health inequalities than baseline factors in older people (60–80 years), However, when risk factors were measured over time, nominal change in explanatory share was relatively larger in the age group over 60 years—with larger increase in explained share by psychosocial and behavioural factors and a larger decrease in explanatory share by biomedical factors and employment in older people compared to the younger population.

In order to diminish possible reversed causality, 9.3% of respondents were excluded from the analyses due to self-reported history of disease. Since these diseases are more prevalent in low socioeconomic positions, a larger group with low education and income has been filtered out. A sensitivity analysis employing all respondents and controlling for morbidity revealed that the adjustment for diseases resulted in smaller socioeconomic inequalities and a slight overestimation of the explanatory share by risk factors. We also conducted a sensitivity analyses excluding persons who died before the second measurement and the results remained the same, with the exception of income inequalities in men`s mortality. Inclusion of men with shorter term survival and income in retirement thus tends to slightly underestimate the income inequalities as well as the potential of their explanation by accounting for change. Another sensitivity analysis restricted only to respondents with complete data conveyed that exclusion of missing categories from the analysis provided similar results to those presented herein.

## Discussion

This study revealed that addition of a second measurement for risk factors provided rather modest improvement in explaining educational and income inequalities in mortality in men and women. Behavioral factors proved to be the most important group of factors in explaining educational inequalities in mortality in men and women (and also income inequalities in women), with largest increase in explained share when measured over time. Psychosocial factors were most important in explaining income inequalities in mortality in men, with very small increase in explanatory share over time. On the contrary, known biomedical factors together with employment status had more predicative power at the baseline compared to measurement over time. These differences in explained share between different groups of factors over time can be attributed to different development in social patterning of explanatory risk factors over time.

Previous research indicates that behavioural factors are among the most important in explaining mortality inequalities [18, 30], and our findings support these conclusions. In particular, our results underscore the importance of behavioural factors for low educated women`s mortality. A comparison with results from British [19], Dutch [3, 5] and French [20] based studies indicates that the effect size of behaviours in explaining mortality inequalities in Norway is between British and French populations and somewhat comparable to Dutch respondents. Our results also highlight the impact of these factors due to increased social patterning over time (especially former smoking and physical inactivity in men, and heavy and former smoking and physical inactivity in low income—but not low educated women). The largest increment in explaining excess mortality due to behavioural change was observed in women with lowest versus highest income. Despite having an egalitarian welfare state [31], Norway`s health promoting messages might have had a larger impact on information uptake and change in unhealthy behaviours in the most affluent groups [32]. Nevertheless, the size of explained share in mortality due to change in behaviour was rather modest in the current inquiry compared to the Whitehall study [18]. We suggest that these differences between the studies may be ascribed to differences in the populations studied, differences in measurements [33], and to differences in culture and social patterning of risk factors [20, 33].

Our findings also underscore the importance of psychosocial factors in health inequalities, comparable in size to other traditional risk factors, such as smoking and overweight [34, 35]. For men, psychosocial factors were much more important for explained share in income and educational inequalities in mortality, than for women. This finding is in line with previous evidence, that the mortality gap between married and unmarried is higher for men [14, 22]. Adverse psychosocial factors in men might be associated with larger exposure to stress and more risky and unhealthy life-styles [36, 37]—and especially in men with low income, since they are more prone to partake in smoking and other risky behaviours as compared to higher income men. To examine this explanation in our data, we calculated the combined explanatory share of psychosocial and behavioural factors at baseline, resulting in odds ratio for the lowest income quartile (OR = 1.29, 95% CI 1.19–1.39) explaining 52% of the excess mortality. The indirect effect of psychosocial factors on men`s mortality through behavioural factors was thus 8%. The effect size of psychosocial factors in men`s mortality is comparable to other studies (which were not gender stratified) [3, 21, 38]. However, change in psychosocial factors did not much amend the explanation of mortalities inequalities—most likely due to decrease in social patterning of psychosocial factors (by income and education) over time. A US-based study showed slightly larger explanatory power of change in psychosocial measures in explaining social inequalities in mortality compared to the present study [21], and such difference might be attributed to differences in included risk factors (e.g. life events).

Biomedical factors and employment status explained rather little in men’s inequalities, but performed better in women at baseline. However, when entered as time-varying variables, the explained part was smaller than at baseline, for both sexes. Our findings correspond with previous research, where repeated measurements of biomedical risk factors improved the explanation of excess risk only to a very little extent [39]. The observed pattern of better explanation by factors measured at baseline can to some extent reflect that biomedical risk factors had generally high prevalence at the baseline in all socioeconomic groups, while differences in prevalence between socioeconomic groups remained the same or reduced. This indicates that in spite of large increase in prevalence of these risk factors in the sample, their minimal social patterning over time did not improve the explanation of inequalities. This is in line with previous findings of decrease in educational disparities in obesity in the US, due to increase in obesity prevalence in all educational groups over time [40]. In addition, socioeconomic inequalities diminish in older populations, most likely due to early death of least healthy individuals [41, 42] and fade-out of biological risk factors in older populations [43]. Since biomedical factors might be the most proximal indicators of physical conditions preceding serious illness/death, it is also reasonable to expect, that these factors can explain mortality better in short term survival than new measurements in people who have survived until follow-up. Similarly, employment status measured earlier in life has perhaps more important bearings for mortality than its change measured 10 years later. In addition, there was an enormous decrease in differences in homemaker employment status between high and low educated women over a 10 years period. In this paper we focused on explaining relative inequalities in mortality, and our rather limited set of biomedical factors seemed to contribute very little to the explanation, however, on the population level, decrease in prevalence of biomedical risks would perhaps result in strong reduction of population health burden in absolute terms [44].

The employed risk factors utilising a single time measurement explained a smaller share of educational inequalities in men`s mortality compared to our previous work utilising the HUNT 2 study [4]. This difference might partly be attributed to different design and a to larger number of explanatory factors employed in the study utilising the mid 90`s data. Although the current study`s results from the mid 80`s are not directly comparable to the results one decade later, they might also reflect the fact of increasing relative educational inequalities in mortality in Norwegian men and women over time [45] as well as a stronger social patterning of lifestyle risk factors, such as smoking [10].

Explanatory factors of mortality inequalities might operate differently in younger as compared to older populations due to differences in social stratification, social distribution of risk factors or health selection [38]. We found that education and income inequalities in mortality were generally smaller in older people. Our finding of larger explanation of health inequalities by baseline factors in younger population compared to older people is in line with prior research [11]. This pattern can perhaps be ascribed to larger mortality inequalities and more pronounced differences in risk factors related to premature mortality [14]. We also found that the explained share by change in psychosocial and behavioural factors and the decrease in explanatory share by change in biomedical factors and employment were larger in the older population as compared to the younger. This finding can be attributed to higher or lower explanatory power of different risk factors of survivors in an older subpopulation with relatively high mortality rates and successively decreasing mortality inequalities, compared to the younger subsample. Nevertheless, research on cumulative risk in elderly population is still very limited [11, 46], as most previous studies focused on middle-aged [8, 20].

### Strengths and Limitations

This study has several advantages, such as examining a large population over several points in time and being the first study to report mortality results separately for men and women in order to gain a better perspective on gender differentials. However, it is not without limitations. A shortcoming to this study is that we included a wide range of factors, however some factors, which have been found to play an important role in explanation of health inequalities in other studies (e.g. material factors, or diet [3, 20]) were beyond measurement possibilities of the current study. Inclusion of other factors or more repeated measurements could potentially provide a slightly different picture [47]. Measurement error introduced by selection of variables and measurement accuracy of those variables together with omitting any potential confounding factors for the exposure—outcome association and explanatory factor-outcome association might have resulted in inaccurate estimation [48]. Our analysis was based on the assumption of causal relations between explanatory risk factors and mortality and no interaction by the explanatory factors [48]. We acknowledge that mediation analysis accounting for exposure-mediation interaction and assessing direct and indirect effects might provide more accurate estimates [48]. In addition, our statistical approach could not account for possible confounding of the direct effect of SES on mortality [49]. It is also known, that explanatory factors are interrelated to each other and can work as mediators [3, 4] as confounders [48], or interact with each other, the latter possibly resulting in increased explanatory share [50, 51]. In order to simplify presentation of the results, we calculated odds ratios only for each set of risk factors separately, although the possible pathways and relations between different groups of risk factors are much more complex and mutual influences cannot be precluded—i.e. the effect of behavioural risk factors might be mediated via psychosocial factors and vice versa. The division of risk factors into groups was based on previous research [4], however, some risk factors could arguably be assigned to another group of risk factors. Moreover, income can also be viewed as an important material factor, through which education might influence mortality. Furthermore, since social patterning of health risk factors may vary between countries, studies of other populations might reach different conclusions.

## Conclusions

This study revealed that inclusion of repeated measurement of risk factors had only modest effect on explaining educational and income inequalities in mortality in Norwegian men and women. Our results highlight the significance of behavioural factors and their socially conditioned patterning in both men and women—in particular between low and high educated women,—with a growing divide in relation to mortality. Findings thus call attention to the importance of health policy measures to alter behavioural paths in socioeconomically disadvantaged groups in order to tackle the growing health gap. Our finding of the relative importance of psychosocial factors in men`s mortality, but with its comparatively small increase in explanation over time, suggests, that the setup of men’s psychosocial factors related to low socioeconomic position represents a considerable risk in respect to mortality, remaining approximately constant over a 10 year period. This implicates that measures to prevent such risks are best to be implemented early in the life course of men. Before reaching a conclusion whether timing of measurement can influence how well different pathways explain socioeconomic inequality in health, more comparative research is required. Nevertheless, we underscore, that although measurement of time-varying risk might increase explained share of health inequalities, other factors, such as employment or biomedical risk, might provide more explanation, when assessed at baseline.
